# Accelerating clinical evidence synthesis with large language models

**DOI:** 10.1038/s41746-025-01840-7

**Published:** 2025-08-08

**Authors:** Zifeng Wang, Lang Cao, Benjamin Danek, Qiao Jin, Zhiyong Lu, Jimeng Sun

**Affiliations:** 1 Siebel School of Computing and Data Science, University of Illinois Urbana-Champaign, Urbana, IL USA; 2 Division of Intramural Research, National Library of Medicine, National Institutes of Health, Bethesda, MD USA; 3 Carle Illinois College of Medicine, University of Illinois Urbana-Champaign, Urbana, IL USA; 4 Present Address: Keiji.AI Inc, Seattle, USA

**Keywords:** Computer science, Medical research, Literature mining

## Abstract

Clinical evidence synthesis largely relies on systematic reviews (SR) of clinical studies from medical literature. Here, we propose a generative artificial intelligence (AI) pipeline named TrialMind to streamline study search, study screening, and data extraction tasks in SR. We chose published SRs to build TrialReviewBench, which contains 100 SRs and 2,220 clinical studies. For study search, it achieves high recall rates (Ours 0.711–0.834 v.s. Human baseline 0.138–0.232). For study screening, TrialMind beats previous document ranking methods in a 1.5–2.6 fold change. For data extraction, it outperforms a GPT-4’s accuracy by 16–32%. In a pilot study, human-AI collaboration with TrialMind improved recall by 71.4% and reduced screening time by 44.2%, while in data extraction, accuracy increased by 23.5% with a 63.4% time reduction. Medical experts preferred TrialMind’s synthesized evidence over GPT-4’s in 62.5%-100% of cases. These findings show the promise of accelerating clinical evidence synthesis driven by human-AI collaboration.

## Introduction

Clinical evidence is crucial for supporting clinical practices and advancing new drug development and needs to be updated regularly^1^. It is primarily gathered through retrospective analysis of real-world data or through prospective clinical trials that assess new interventions on humans. Researchers usually conduct systematic reviews to consolidate evidence from various clinical studies in the literature^2,3^. However, this process is expensive and time-consuming, requiring an average of five experts and 67.3 weeks based on an analysis of 195 systematic reviews^4^. Moreover, the fast growth of clinical study databases means that the information in these published clinical reviews becomes outdated rapidly^5^. For instance, PubMed has indexed over 35M citations and gets over 1M new citations annually^6^. This situation underscores the urgent need to streamline the systematic review processes to document systematic and timely clinical evidence from the extensive medical literature^1,7^.

Large language models (LLMs) excel at instruction following: they can perform target tasks with the task definition and examples as the text inputs (namely “prompts”)^8^. Recent works have hence adopted LLMs for various systematic review and meta-analysis tasks, such as generating searching queries^9,10^, extracting studies’ attributes^11–14^, screening citations^15–17^, and summarizing findings from multiple studies^18–21^. However, few have investigated LLMs’ effectiveness across the evidence synthesis process as outlined by standard practice such as PRISMA statement^22–25^. An understanding of the strengths and limitations of LLMs in practical systematic literature review and meta-analysis tasks is crucial for their development. To fill this gap, we created a testing dataset TrialReviewBenchbased on 100 published systematic literature reviews with 2220 associated clinical studies. To align with key steps in the PRISMA statement, we built testing tasks for study search, citation screening, and data extraction. It also consists of manual annotations of 1334 study characteristics and 1049 study results.

This study aims to further fill the gap in adapting LLMs to evidence synthesis tasks, primarily overcoming LLM’s limitations in (1) hallucinations, (2) weakness in reasoning with numerical data, (3) overly generic outputs, and (4) lack of transparency and reliability^26^. Specifically, we developed an AI-driven pipeline named TrialMind, which is optimized for (1) generating boolean queries to search citations from the literature; (2) building eligibility criteria and screening through the found citations; and (3) extracting data, including study protocols, methods, participant baselines, study results, etc., from publications and reports. More importantly, TrialMind breaks down into subtasks that adhere to the established practice of systematic reviews^23^, which facilitates experts in the loop to monitor, edit, and verify intermediate outputs. It also has the flexibility to allow experts to begin at any intermediate step as needed.

In this study, we show that the TrialMind can (1) retrieve a complete list of target studies from the literature, (2) follow the specified eligibility criteria to rank the most relevant studies at the top, and (3) achieve high accuracy in extracting information and clinical outcomes from unstructured documents based on user requests. Beyond providing descriptive evidence, TrialMind can extract numerical clinical outcomes to be standardized as input for meta-analysis (e.g., forest plots). A human evaluation was conducted to assess the synthesized evidence. Finally, to validate the practical benefits, we developed an accessible web application based on TrialMind and conducted a user study comparing two approaches: AI-assisted experts versus standalone experts. We measured the time savings and evaluated the output quality of each approach. The results show that TrialMind significantly reduced the time required for study search, citation screening, and data extraction, while maintaining or improving the quality of the output compared to experts working alone.

## Results

### Creating TrialReviewBench from medical literature

In this study, we aim to create a practical benchmark using published systematic reviews. Given a review paper, the included studies are extracted as target studies for identification and screening, while data from the study characteristics tables serve as ground truth for data extraction. This setup ensures evaluation accuracy and alignment with PRISMA practices. An illustration of this process is in Supplementary Fig. 1b. Specifically, we retrieved a list of cancer treatments from the National Cancer Institute’s introductory page as the keywords to search medical systematic reviews^27^. To ensure data quality, we crafted comprehensive queries with automatic filtering and manual screening. For each review, we obtained the list of studies with their PubMed IDs, retrieved their full content, and extracted study characteristics and clinical outcomes. We followed PubMed’s usage policy and guidelines during retrieval. Further manual checks were performed to correct inaccuracies, eliminate invalid and duplicate papers, and refine the text for clarity (“Methods”). The final TrialReviewBenchdataset consists of 2220 studies involved in 100 reviews (Fig. 2a), covering four major therapy topics: Immunotherapy, Radiation/Chemotherapy, Hormone Therapy, and Hyperthermia. We manually created three major evaluation tasks based on these reviews: study search, study screening, and data extraction.

The study search task begins with the PICO (Population, Intervention, Comparison, Outcome) elements defined by the selected systematic review, which serve as the formal definition of the research question. The model being tested is tasked with generating keywords for the treatment and condition terms, as depicted in Fig. 2e. These keywords are then used to form Boolean queries, which are submitted to search citations in the PubMed database. The Recall performance of the model is evaluated by checking whether the retrieved studies include those that were actually involved in the systematic review.

For the citation screening task, we mixed the ground truth studies into the search results to create a candidate set of 2000 citations. The model being tested ranks these citations based on the likelihood that each citation should be included in the systematic review. To assess the model’s performance, we compute Recall@*k*: the recall value indicating how many of the ground truth studies appear in the top *k* ranked candidates.

The data extraction test set was built based on the study characteristics table from each systematic review, which typically details study characteristics such as study design, population demographics, and outcome measurements (Supplementary Fig. 1). We manually extracted the values to create 1334 study characteristic annotations. Additionally, we extract individual study results from the review’s reported analysis, often presented in forest plots, capturing metrics such as overall response and event rates, resulting in 1049 study result annotations. We evaluate model accuracy by manually checking the extracted values against the ground truth values extracted from the systematic reviews for each involved study.

### Build an LLM-driven system for clinical evidence synthesis

**Fig. 1:**
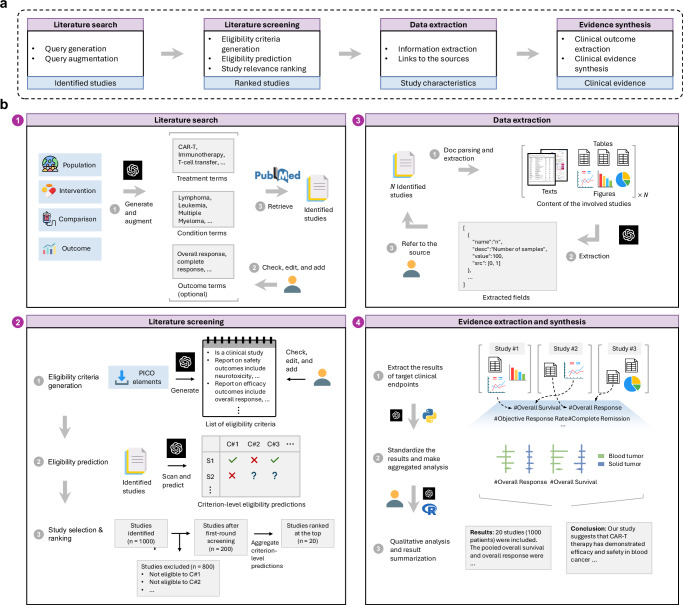
The overview of TrialMind pipeline. **a** It has four main steps: literature search, literature screening, data extraction, and evidence synthesis. **b** (1) Utilizing input PICO as the review objective, TrialMind generates search terms to identify studies from literature databases. (2) TrialMind suggests the inclusion and exclusion criteria for users to check and edit. Then, TrialMind scans all the identified studies and assesses each individual’s eligibility for each criterion. Last, the user decides on the final strategy to aggregate the criterion-level assessments into an overall eligibility score to rank the eligibility of all identified studies. (3) Given the defined data field, TrialMind processes the content of the involved studies and produces the structured outputs for each data field, grounded on the source indices for user inspection. (4) TrialMind takes the extracted data to create the standardized trial outcomes and works with users to aggregate all involved trial outcomes with a meta-analysis to create new clinical evidence.

We develop TrialMind to seamlessly integrate into the PRISMA workflow for systematic literature reviews in medicine. According to the PRISMA flowchart, the process consists of three main stages: (1) identification, (2) screening, and (3) inclusion. As illustrated in Supplementary Fig. 1a, TrialMind aligns with PRISMA by streamlining these stages-generating search terms from PICO elements, applying inclusion and exclusion criteria for eligibility assessment, and extracting target data fields while aggregating study outcomes via meta-analysis. This design enhances efficiency in systematic literature reviews while supporting human-AI collaboration (Fig. 1 and Methods).

### TrialMind can make a comprehensive retrieval of studies from the literature

**Fig. 2:**
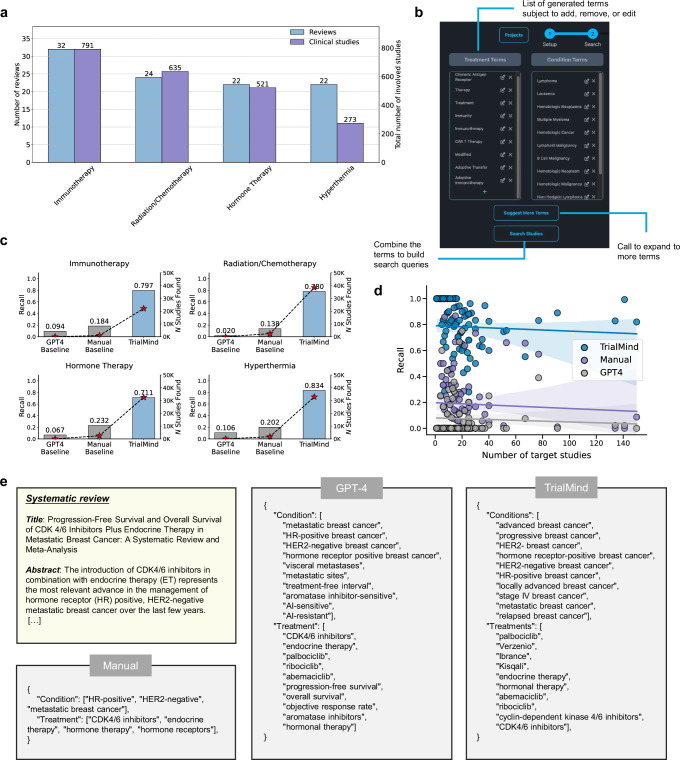
Literature search experiment results. **a** The distribution of systematic literature reviews and clinical studies in the evaluation dataset categorized by the primary types of investigated interventions. **b** The TrialMind interface for literature search allows users to refine search terms efficiently. TrialMind suggests an initial set of treatment and condition terms, which users can modify by adding, editing, or removing terms. A sampling button enables users to explore additional terms generated by TrialMind. Once the term set is finalized, users can click the “Search Studies” button to construct the search query and retrieve relevant studies from the literature. **c** The recall of search results for reviews across four topics. The left y-axis represents the average recall achieved by different methods, while the right y-axis shows the number of identified studies. TrialMind successfully captures 70–80% of ground truth studies, whereas other methods miss most of them. **d** Scatter plots of the Recall against the number of ground truth studies. It is assumed that the more ground truth studies there are, the harder it is to cover all of them via one attempted search query. Each scatter indicates the results of one review. Regression estimates are displayed with the 95% CIs in blue or purple. TrialMind shows a consistent superiority over the other methods while maintaining invariance to the increasing complexity of the searching task. **e** Example cases comparing the outputs of three methods. The manually crafted terms are usually precise while not diverse enough to cover all the variants in the literature studies. A vanilla GPT-4 approach can introduce terms that are either too broad or irrelevant to the research objective.

Finding relevant studies from medical literature like PubMed, which contains over 35 million entries, can be challenging. Typically, this requires the research expertise to craft complex queries that comprehensively cover pertinent studies. The challenge lies in balancing the specificity of queries: too stringent, and the search may miss relevant studies; too broad, and it becomes impractical to manually screen the overwhelming number of results. Previous approaches propose to prompt LLMs to generate the searching query directly^9^, which can induce incomplete searching results due to the limited knowledge of LLMs. In contrast, TrialMind is designed to produce comprehensive queries through a pipeline that includes query generation, augmentation, and refinement. It also provides users with the ability to make further adjustments (Fig. 2b).

The dataset involving clinical studies spanning ten cancer treatment areas was used for evaluation (Fig. 2a). For each review, we collected the involved studies’ PubMed IDs as the ground truth and measured the Recall, i.e., how many ground truth studies are found in the search results. We created two baselines as the comparison: GPT-4 and Human. The GPT-4 baseline makes a prompt for LLMs to generate the boolean queries^9^. It represents the common way of prompting LLMs for literature search query generation. The Human baseline represents a way where the key terms from PICO elements are extracted manually and expanded, referring to UMLS^28^, to construct the search queries.

Overall, TrialMind achieved a Recall of 0.782 on average for all reviews, meaning it can capture most of the target studies. By contrast, the GPT-4 baseline yielded Recall = 0.073, and the Human baseline yielded Recall = 0.187. We divided the search results across four topics determined by the treatments studied in each review (Fig. 2c). Our analysis showed that TrialMind can identify many more studies than the baselines. For instance, TrialMind achieved Recall = 0.797 with identified studies *N* = 22,084 for Immunotherapy-related reviews, while the GPT-4 baseline got Recall = 0.094 (*N* studies = 27), and the Human baseline got Recall = 0.154 (*N* studies = 958), respectively. In Radiation/Chemotherapy, TrialMind achieved Recall = 0.780, the GPT-4 baseline got Recall = 0.020, and the Human baseline got Recall = 0.138. In Hormone Therapy, TrialMind achieved Recall = 0.711, the GPT-4 baseline got Recall = 0.067, and the Human baseline got Recall = 0.232. In Hyperthermia, TrialMind achieved Recall = 0.834, the GPT-4 baseline got Recall = 0.106, and the Human baseline got Recall = 0.202. These results demonstrate that regardless of the search task’s complexity, as indicated by the variability in the Human baseline, TrialMind consistently retrieves nearly all target studies from the PubMed database. We have also tested TrialMind in broad therapeutic areas other than oncology. The results can be found in Supplementary Fig. 2.

Furthermore, we generated scatter plots of Recall versus the number of target studies for each review (Fig. 2d). The rationale behind this is that a higher number of ground truth studies to identify generally makes the task more challenging, as it becomes more difficult to construct a perfect search query that captures all relevant studies. The results reveal that TrialMind consistently maintained a high Recall, significantly outperforming the best baselines across all 100 reviews. A trend of declining Recall with an increasing number of target studies was confirmed through regression analysis. It was found that the GPT-4 baseline struggled, showing Recall close to 0, and the Human baseline results varied, with most reviews below 0.5. As the number of target studies increased, the Human and GPT-4 baselines’ Recall decreased to nearly zero. In contrast, TrialMind demonstrated remarkable resilience, showing minimal variation in performance despite the increasing number of target studies. For instance, in a review involving 141 studies, TrialMind achieved a Recall of 0.99, while the GPT-4 and Human baselines obtained a Recall of 0.02 and 0, respectively.

### TrialMind enhances citation screening and ranking

**Fig. 3:**
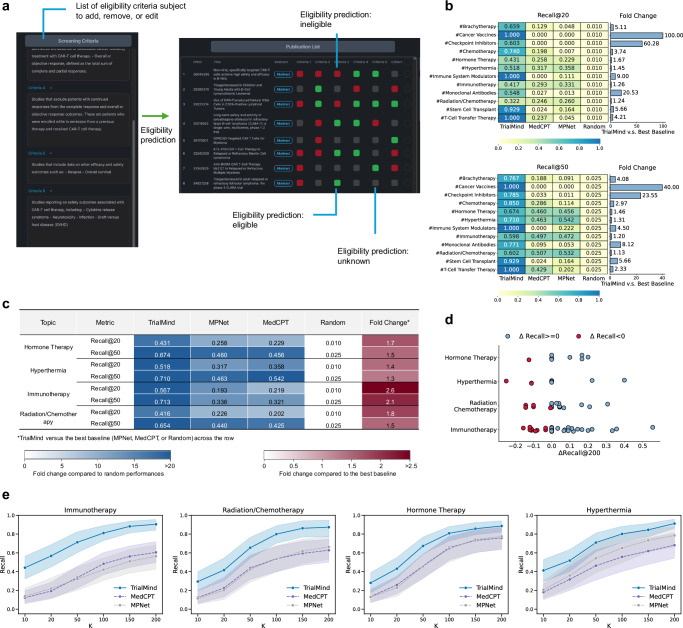
Literature screen experiment results. **a** Streamline study screening using TrialMind with human in the loop. The left panel shows the list of eligibility criteria suggested by TrialMind and is subject to the user’s edits. The right panel shows the TrialMind assessments for the criterion-level eligibility of all identified studies. Red, green, and gray fields indicate the assessment “ineligible”, “eligible”, and “unknown”, respectively. **b** Ranking performances for Recall@20/50 within across therapeutic areas. The bars on the right show the numbers of the fold TrialMind's performance against the best baseline across that row. **c** Recall@20 and Recall@50 for TrialMind and selected baselines. **d** Effect of individual criteria on ranking results. To assess this effect, we remove one criterion at a time from the criteria set, re-rank the results, and measure the change in recall, reflecting the criterion’s impact on ranking quality. Most criteria positively influence the ranking, while a small portion has a negative effect. **e** Ranking performance for Recall*@K* with varying *K* in four topics. Shaded areas are 95% confidence interval.

As in the PRISMA statement for systematic literature review^23^, researchers need to manually create a set of inclusion and exclusion criteria, and then sift through hundreds to thousands of identified studies to assess individual studies’ eligibility to be included in the review. TrialMind streamlines this task through a three-step approach: (1) it generates a set of eligibility criteria based on the research question in PICO format; (2) it applies these criteria to evaluate the study’s eligibility, denoted by {−1, 0, 1} where −1 and 1 represent eligible and non-eligible, and 0 represents unknown/uncertain, respectively; and (3) it ranks the studies by aggregating the eligibility predictions (Fig. 3a). We took a summation of the criteria-level eligibility predictions as the study-level relevance prediction scores for ranking.

We chose MPNet^29^ and MedCPT^30^ as the general domain and medical domain ranking baselines, respectively. These methods compute study relevance by the semantic similarity between the encoded PICO elements and the encoded study’s abstracts. We also set a Random baseline that randomly samples from candidates. For each review, we mixed the target studies with the other found studies to build a candidate set of 2,000 studies for ranking. Discriminating the target studies from the other candidates is challenging since all candidates meet the search queries, meaning they most probably investigate the relevant therapies or conditions. We evaluated the ranking performance using the Recall@20 and Recall@50 metrics.

We found that TrialMind greatly improved ranking performances, with the fold changes over the best baselines ranging from 1.3 to 2.6 across four topics (Fig. 3c). For instance, for the Hormone Therapy topic, TrialMind obtained Recall*@*20 = 0.431 and Recall*@*50 = 0.674. In the Hyperthermia topic, TrialMind obtained Recall*@*20 = 0.518 and Recall*@*50 = 0.710. In the Immunotherapy topic, TrialMind obtained Recall*@*20 = 0.567 and Recall*@*50 = 0.713. In the Radiation/Chemotherapy topic, TrialMind obtained Recall*@*20 = 0.416 and Recall*@*50 = 0.654. In contrast, other baselines exhibit significant variability across different topics. The general domain baseline MPNet was the worst as it performed similarly to the Random baseline in Recall*@*20. MedCPT showed marginal improvement over MPNet in the last three topics, while both failed to capture enough target studies in all topics. We have also tested TrialMind in 16 broad therapeutic areas other than oncology. The results can be found in Supplementary Fig. 2.

Furthermore, TrialMind demonstrated significant improvements over the baselines across various therapeutic areas (Fig. 3b). For example, in “Cancer Vaccines” and “Hormone Therapy,” TrialMind substantially increased Recall*@*50, achieving 33.33-fold and 10.53-fold improvements, respectively, compared to the best-performing baseline. TrialMind generally attained a fold change greater than 2 (ranging from 1.57 to 33.33). Despite the challenge of selecting from a large pool of candidates (*n* = 2, 000) where candidates were very similar, TrialMind identified an average of 43% of target studies within the top 50. We compared TrialMind to MedCPT and MPNet for Recall*@K* (*K* in 10 to 200) to gain insight into how *K* influences the performances (Fig. 3e). We found TrialMind can capture most of the target studies (over 80%) when *K* = 100.

To thoroughly assess the quality of these criteria and their impact on ranking performance, we conducted a leave-one-out analysis to calculate ΔRecall*@*200 for each criterion (Fig. 3d). The ΔRecall*@*200 metric measures the difference in ranking performance with and without a specific criterion, with a larger value indicating superior criterion quality. Our findings revealed that most criteria positively influenced ranking performances, as the negative influence criteria are *n* = 1 in Hormone Therapy, *n* = 1 in Hyperthermia, *n* = 5 in Radiation/Chemotherapy, and *n* = 7 in Immunotherapy. Additionally, we identified redundancies among the generated criteria, as those with ΔRecall*@*200 = 0 were the most frequently observed. This redundancy likely stems from some criteria covering similar eligibility aspects, thus not impacting performance when one is omitted.

### TrialMind scales data and result extraction from publications

**Fig. 4:**
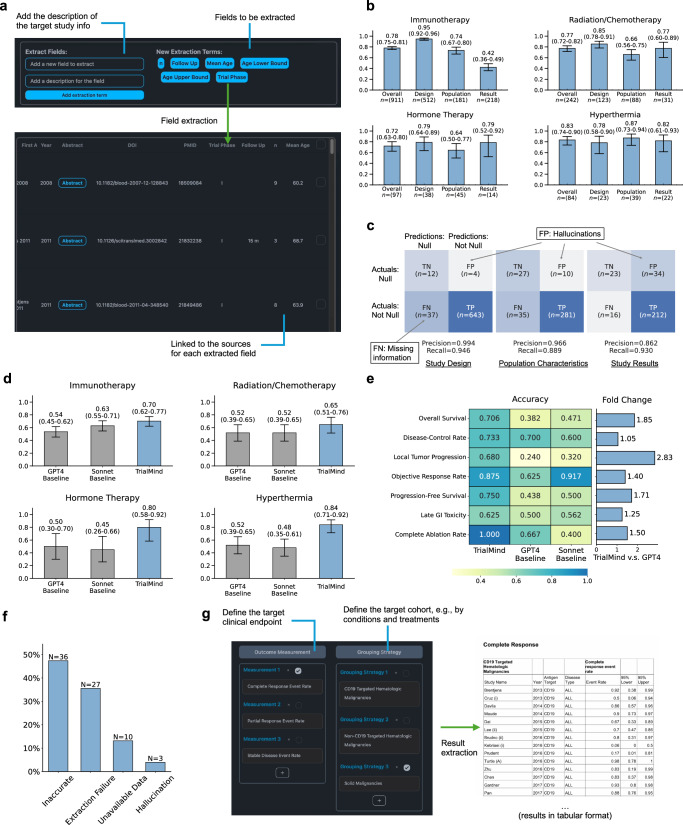
Data extraction experiment results. **a** In the TrialMind platform, users can define the name and description of target data fields and create a structured list. Upon clicking the “Extraction” button, TrialMind processes the selected studies and populates the study table with new columns representing the specified fields. Users can also click on extracted values to view the source text referenced by TrialMind. **b** Accuracy of data extraction across four therapy topics, with results further stratified by data type. **c** Confusion matrix illustrating hallucination and missing rates in data extraction across three data types. **d** Comparison of trial result extraction accuracy between TrialMind and baseline methods across four topics. **e** Comparison of trial result extraction accuracy between TrialMind and baselines across the most frequent clinical endpoint types. **f**, Error analysis of result extraction: Inaccurate—incorrect data extraction; Extraction Failure—TrialMind failed to extract data and returned null; Unavailable Data - target data was absent in the input document, resulting in null output; Hallucination—TrialMind generated data that did not exist in the input document. **g** In TrialMind's result extraction step, users can define the target clinical endpoint and cohort based on conditions and treatments. Clicking the extraction button triggers the extraction process for all selected studies, with results exportable in tabular format for further analysis.

TrialMind streamlines data extraction, such as target therapies, study arm design, and participants’ baseline information from involved studies. Specifically, TrialMind refers to the field names and the descriptions from users and use the full content of the study documents in PDF or XML formats as inputs (Fig. 4a). When the free full content is unavailable, TrialMind accepts the user-uploaded content as the input. We developed an evaluation dataset by converting the study characteristic tables from each review paper into data points. Our dataset comprises 1,334 target data points, including 696 on study design, 353 on population features, and 285 on results. We assessed the data extraction performance using the Accuracy metric.

TrialMind demonstrated strong extraction performance across various topics (Fig. 4b): it achieved an accuracy of ACC = 0.78 (95% confidence interval (CI) = 0.75–0.81) in the Immunotherapy topic, ACC = 0.77 (95% CI = 0.72–0.82) in the Radiation/Chemotherapy topic, ACC = 0.72 (95% CI = 0.63–0.80) in the Hormone Therapy topic, and ACC = 0.83 (95% CI = 0.74–0.90) in the Hyperthermia topic. These results indicate that TrialMind can provide a solid initial data extraction, which human experts can refine. Importantly, each output can be cross-checked by the linked original sources, facilitating verification and further investigation.

Diving deeper into the accuracy across different types of fields, we observed varying performance levels. It performed best in extracting study design information, followed by population details, and showed the lowest accuracy in extracting results (Fig. 4b). For example, in the Immunotherapy topic, TrialMind achieved an accuracy of ACC = 0.95 (95% CI = 0.92–0.96) for study design, ACC = 0.74 (95% CI = 0.67–0.80) for population data, and ACC = 0.42 (95% CI = 0.36–0.49) for results. This variance can be attributed to the prevalence of numerical data in the fields: fields with more numerical data are typically harder to extract accurately. Study design is mostly described in textual format and is directly presented in the documents, whereas population and results often include numerical data such as the number of patients or gender ratios. Results extraction is particularly challenging, often requiring reasoning and transformation to capture values accurately. Given these complexities, it is advisable to scrutinize the extracted numerical data more carefully.

We also evaluated the robustness of TrialMind against hallucinations and missing information (Fig. 4c). We constructed a confusion matrix detailing instances of hallucinations: false positives (FP) where TrialMind generated data not present in the input document, and false negatives (FN) where it failed to extract available target field information. We observed that TrialMind achieved a precision of Precision = 0.994 for study design, Precision = 0.966 for population, and Precision = 0.862 for study results. Missing information was slightly more common than hallucinations, with TrialMind achieving recall rates of Recall = 0.946 for study design, Recall = 0.889 for population, and Recall = 0.930 for study results. The incidence of both hallucinations and missing information was generally low. However, hallucinations were notably more frequent in study results; this often occurred because LLMs could confuse definitions of clinical outcomes, for example, mistaking ‘overall response’ for ‘complete response.’ Nevertheless, such hallucinations are typically manageable, as human experts can identify and correct them while reviewing the referenced material.

The challenges in extracting study results primarily stem from (1) identifying the locations that describe the desired outcomes from lengthy papers, (2) accurately extracting relevant numerical values such as patient numbers, event counts, durations, and ratios from the appropriate patient groups, and (3) performing the correct calculations to standardize these values for meta-analysis. In response to these complexities, we developed a specialized pipeline for result extraction (Fig. 4g), where users provide the interested outcome and the cohort definition. TrialMind offers a transparent extraction workflow, documenting the sources of results along with the intermediate reasoning and calculations.

We compared TrialMind against two generalist LLM baselines, GPT-4 and Sonnet, which were prompted to extract the target outcomes from the full content of the study documents. Since the baselines can only make text extractions, we manually convert them into numbers suitable for meta-analysis^31^. This made very strong baselines since they combined LLM extraction with human post-processing. We assessed the performance using the Accuracy metric.

The evaluation conducted across four topics demonstrated the superiority of TrialMind (Fig. 4d). Specifically, in Immunotherapy, TrialMind achieved an accuracy of ACC = 0.70 (95% CI 0.62–0.77), while GPT-4 scored ACC = 0.54 (95% CI 0.45–0.62). In Radiation/Chemotherapy, TrialMind reached ACC = 0.65 (95% CI 0.51–0.76), compared to GPT-4’s ACC = 0.52 (95% CI 0.39–0.65). For Hormone Therapy, TrialMind achieved ACC = 0.80 (95% CI 0.58–0.92), outperforming GPT-4, which scored ACC = 0.50 (95% CI 0.30–0.70). In Hyperthermia, TrialMind obtained an accuracy of ACC = 0.84 (95% CI 0.71–0.92), significantly higher than GPT-4’s ACC = 0.52 (95% CI 0.39–0.65). The breakdowns of evaluation results by the most frequent types of clinical outcomes (Fig. 4e) showed TrialMind got fold changes in accuracy ranging from 1.05 to 2.83 and a median of 1.50 over the best baselines. This enhanced effectiveness is largely attributable to TrialMind’s ability to accurately identify the correct data locations and apply logical reasoning, while the baselines often produced erroneous initial extractions.

We analyzed the error cases in our result extraction experiments and identified four primary error types (Fig. 4f). The most common error was ‘Inaccurate’ extraction (n=36), followed by ‘Extraction failure’ (*n* = 27), “Unavailable data” (*n* = 10), and ‘Hallucinations’ (*n* = 3). ‘Inaccurate’ extractions often occurred due to multiple sections ambiguously describing the same field. For example, a clinical study might report the total number of participants receiving CAR-T therapy early in the document and later provide outcomes for a subset with non-small cell lung cancer (NSCLC). The specific results for NSCLC patients are crucial for reviews focused on this subgroup, yet the presence of general data can lead to confusion and inaccuracies in extraction. ‘Extraction failure’ and ‘Unavailable data’ both illustrate scenarios where TrialMind could not retrieve the information. The latter case particularly showcases TrialMind’s robustness against hallucinations, as it failed to extract data outside the study’s main content, such as in appendices, which were not included in the inputs. Furthermore, errors caused by hallucinations were minor. The outputs were easy to identify and correct through manual inspection since no references were provided.

### TrialMind facilitates clinical evidence synthesis via human-AI collaboration

**Fig. 5:**
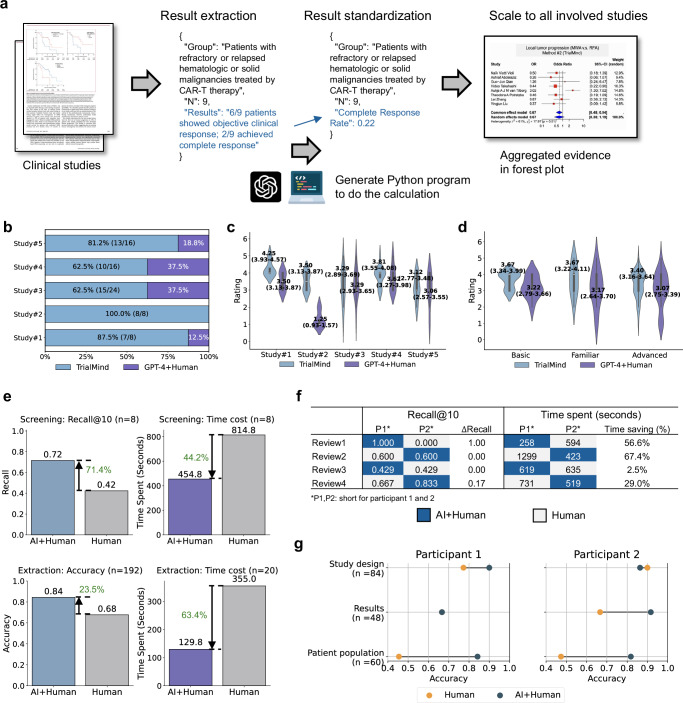
Results of human evaluation and user study. **a** TrialMind's systematic result extraction pipeline. The process begins with an input study document, from which TrialMind generates a focused summary of the target group’s main results. It then systematically extracts key numerical data and performs programmatic calculations to standardize the results. **b** A comparative evaluation where eight expert annotators assessed the quality of meta-analysis results between TrialMind and a baseline consisting of GPT-4 extraction with human post-processing. The analysis presents TrialMind's winning rate relative to the baseline across five selected systematic reviews. **c** Violin plots illustrating annotator ratings distribution for meta-analysis results, comparing TrialMind against the GPT-4 baseline across five systematic reviews. Each plot displays the mean ratings with 95% confidence intervals derived from all annotator assessments. **d**, Violin plots showing rating distributions stratified by annotators' self-reported expertise levels. Each plot indicates the mean ratings with 95% confidence intervals aggregated across all evaluated studies. **e** Comparative analysis of two experimental arms: Human (manual effort) versus AI+Human (TrialMind-assisted). The upper panel presents the overall Recall achieved by two human participants when screening 100 identified studies, alongside time duration. The lower panel displays the overall Accuracy achieved in data extraction tasks, with corresponding time measurements for both arms. **f** Detailed breakdown of screening performance metrics across different systematic reviews and participants, comparing AI+Human and Human arms. **g** Comprehensive comparison of data extraction accuracy between AI+Human and Human arms, analyzed across different participants and data types.

We selected five systematic review studies as benchmarks and referenced the clinical evidence reported in the target studies. The baseline used GPT-4 with a simple prompting to extract the relevant text pieces that report the target outcome of interest (Methods). Manual calculations were necessary to standardize the data for meta-analysis. In contrast, TrialMind automated the extraction and standardization (Fig. 5a by (1) extracting the raw result description from the input document and (2) standardizing the results by generating a Python program to assist the calculation. The standardized results from all involved studies are then fed into the R program by human experts to make the aggregated evidence in a forest plot.

We engaged with eight annotators (five medical doctors and three computer scientists) to assess the quality of synthesized clinical evidence presented in forest plots. Each annotator was asked to evaluate the evidence quality by comparing it against the evidence reported in the target review and deciding which method, TrialMind or the baseline, produced superior results (Supplementary Fig. 3). Additionally, they rated the quality of the synthesized clinical evidence on a scale of 1 to 5. The assignment of our method and the baseline was randomized to ensure objectivity. The results highlighted TrialMind’s superior performance compared to the direct use of GPT-4 for clinical evidence synthesis (Fig. 5b). We calculated the winning rate of TrialMind versus the baseline across the five studies. The results indicate a consistent preference by annotators for the evidence synthesized by TrialMind over that of the baseline. Specifically, TrialMind achieved winning rates of 87.5%, 100%, 62.5%, 62.5%, and 81.2%, respectively. The baseline’s primary shortcoming stemmed from the initial extraction step, where GPT-4 often failed to identify the relevant sources without well-crafted prompting. Therefore, the subsequent manual post-processing was unable to rectify these initial errors.

In addition, we illustrated the ratings of TrialMind and the baseline across studies (Fig. 5c). We found TrialMind was competent as the GPT-4+Human baseline and outperformed the baseline in many scenarios. For example, TrialMind obtained the mean rating of 4.25 (95% CI 3.93–4.57) in Study #1 while the baseline obtained 3.50 (95% CI 3.13–3.87). In Study #2, TrialMind yielded 3.50 (95% CI 3.13–3.87) while the baseline yielded 1.25 (95% CI 0.93–1.57). The performance of the two methods was comparable in the remaining three studies. These results highlight TrialMind as a highly effective approach for streamlining data extraction and processing while maintaining the critical benefit of human oversight.

We requested that annotators self-assess their expertise level in clinical studies, classifying themselves into three categories: “Basic”, “Familiar”, and “Advanced”. The typical profile ranges from computer scientists at the basic level to medical doctors at the advanced level. We then analyzed the ratings given to both methods across these varying expertise levels (Fig. 5d). We consistently observed higher ratings for TrialMind than the baseline across all groups. Annotators with basic knowledge tended to provide more conservative ratings, while those with more advanced expertise offered a wider range of evaluations. For instance, the “Basic” group provided average ratings of 3.67 (95% CI 3.34–3.39) for TrialMind compared to 3.22 (95% CI 2.79–3.66) for the baseline. The “Advanced” group rated TrialMind at an average of 3.40 (95% CI 3.16–3.64) and the baseline at 3.07 (95% CI 2.75–3.39).

We conducted user studies to compare the quality and time efficiency between purely manual efforts and human-AI collaboration using TrialMind. Two participants were involved in both study screening and data extraction tasks. For the screening task, each participant was assigned 4 systematic review papers, with 100 candidate citations identified for each review. The participants were asked to select the 10 most likely relevant citations from the candidate pool. Each participant was provided with 2 candidate sets pre-ranked by TrialMind and 2 unranked sets. The participants also recorded the time taken to complete the screening process for each set. For the data extraction task, each participant was given 10 clinical studies. They manually extracted the target information for 5 of these studies. For the other 5, TrialMind was first used to perform an initial extraction, and the participants were required to verify and correct the extracted results. The time taken for the extraction process was reported for each study.

In Fig. 5e, we present the average performance and time cost for the AI+Human and Human-only approaches across both the study screening and data extraction tasks. The results demonstrate that the AI+Human approach consistently outperforms the Human-only approach. For the screening tasks, AI+Human achieved a 71.4% relative improvement in Recall while reducing time by 44.2% compared to the Human-only arm. This underscores the significant advantage of TrialMind in accelerating the study screening process while also improving its quality. Similarly, for the data extraction tasks, the AI + Human approach improved extraction accuracy by 23.5% on average, with a 63.4% reduction in time required.

Detailed results of screening time and performance are shown in Fig. 5f, where two reviews showed the AI+Human approach achieving the same Recall as the Human-only arm with notable time savings, and in two other reviews, AI + Human achieved higher Recall with less time. From Fig. 5g, we see that the AI+Human approach delivered better or comparable accuracy across all three types of data, with the smallest gap in “Study design”. This is likely because study design information is often readily available in the study abstract, making it relatively easier for humans to extract. In contrast, the other two data types are embedded deeper within the main content, which can sometimes make it challenging for human readers to locate the correct information.

## Discussion

Clinical evidence forms the bedrock of evidence-based medicine, crucial for enhancing healthcare decisions and guiding the discovery and development of new therapies. It often comes from a systematic review of diverse studies found in the literature, encompassing clinical trials and retrospective analyses of real-world data. Yet, the burgeoning expansion of literature databases presents formidable challenges in efficiently identifying, summarizing, and maintaining the currency of this evidence. For instance, a study by the US Agency for Healthcare Research and Quality (AHRQ) found that half of 17 clinical guidelines became outdated within a couple of years^32^.

The rapid development of large language models (LLMs) and AI technologies has generated considerable interest in their potential applications in clinical research^33,34^. However, most of them focused on an individual aspect of the clinical evidence synthesis process, such as literature search^35,36^, citation screening^37–39^, quality assessment^40^, or data extraction^41,42^. In addition, implementing these models in a manner that is collaborative, transparent, and trustworthy poses significant challenges, especially in critical areas such as medicine^43^. For instance, when utilizing LLMs to summarize evidence from multiple studies, the descriptive summaries often usually merely echo the findings verbatim, omit crucial details, and fail to adhere to established best practices^18^. Besides, when given a set of studies that are irrelevant to the research question, LLMs are prone to produce hallucinations and hence cause misleading evidence^44^. This challenge highlights the need for an integrated pipeline that is aligned with standard practice, such as PRISMA for systematic literature reviews to strategically pick the target studies for analysis^45,46^, or enhanced with human-AI collaboration^47^.

This study introduces a clinical evidence synthesis pipeline enhanced by LLMs, named TrialMind. This pipeline is structured in accordance with established medical systematic review protocols such as PRISMA, involving steps such as study searching, screening, data/result extraction, and evidence synthesis. At each stage, human experts have the capability to access, monitor, and modify intermediate outputs. This human oversight helps to eliminate errors and prevents their propagation through subsequent stages. Unlike approaches that solely depend on the knowledge of LLMs, TrialMind integrates human expertise through in-context learning and chain-of-thought prompting. Additionally, TrialMind extends external knowledge sources to its outputs through retrieval-augmented generation and leveraging external computational tools to enhance the LLM’s reasoning and analytical capabilities. Comparative evaluations of TrialMind and traditional LLM approaches have demonstrated the advantages of this system design in LLM-driven applications within the medical field.

This study also has several limitations. First, despite incorporating multiple techniques, LLMs may still make errors at any stage. Therefore, human oversight and verification remain crucial when implementing TrialMind in practical settings. Second, while TrialMind demonstrated effectiveness in study search, screening, and data extraction, the dataset used was limited in size due to the high costs associated with human labeling. Specifically, TrialMind was evaluated on clinical trials and observational studies primarily in oncology and focused on therapeutic outcomes, its generalizability to broader domains, such as preventive interventions, diagnostics, or non-oncology topics, remains to be established. Third, the study coverage was restricted to publicly available sources from PubMed Central, which provides structured PDFs and XMLs. Many relevant studies are either not available on PubMed or are in formats that entail OCR algorithms as preprocessing, indicating a need for further engineering to incorporate broader data sources. Fourth, although TrialMind illustrated the potential of using advanced LLMs like GPT-4 to streamline clinical evidence synthesis, it is not yet an end-to-end solution for all steps in systematic literature reviews. Future development for several other steps, such as study quality assessment and report drafting, can further increase its value. Last, while the use of LLMs like GPT-4 can accelerate study screening and data extraction, the associated costs and processing times may present bottlenecks in some scenarios. Future enhancements that improve efficiency or utilize localized, specialized models could increase practical utility.

LLMs have made significant strides in AI applications. TrialMind exemplifies a crucial aspect of system engineering in LLM-driven pipelines, facilitating the practical, robust, and transparent use of LLMs. We anticipate that TrialMind will benefit the medical AI community by fostering the development of LLM-driven medical applications and emphasizing the importance of human-AI collaboration.

## Methods

### Description of the TrialReviewBench dataset

We present the overall flow in building the TrialReviewBenchdata in Supplementary Fig. 4.

For database search and initial filtering, we undertook a comprehensive search on the PubMed database for meta-analysis papers related to cancer. The Boolean search terms were specifically chosen to encompass a broad spectrum of cancer-related topics. These terms included “cancer”, “oncology”, “neoplasm”, “carcinoma”, “melanoma”, “leukemia”, “lymphoma”, and “sarcoma”. Additionally, we incorporated terms related to various treatment modalities such as “therapy”, “treatment”, “chemotherapy”, “radiation therapy”, “immunotherapy”, “targeted therapy”, “surgical treatment”, and “hormone therapy”. To ensure that our search was exhaustive yet precise, we also included terms like “meta-analysis” and “systematic review” in our search criteria.

This initial search yielded an extensive pool of 46,192 results, reflecting the vast research conducted in these areas. We applied specific filters to refine these results and ensure relevance and quality. We focused on articles where PMC Full text was available and specifically categorized under “Meta-Analysis”. Further refinement was done by restricting the time frame of publications to those between January 1, 2020, and January 1, 2023. We also narrowed our focus to studies conducted on humans and those available in English. This filtration process leads to an initial collection of 2691 reviews.

Building upon our initial search, we employed further refinement techniques using both MeSH terms and specific keywords. The MeSH terms were carefully selected to target papers precisely relevant to various forms of cancer. These terms included “cancer”, “tumor”, “neoplasms”, “carcinoma”, “myeloma”, and “leukemia”. This focused approach using MeSH terms effectively reduced our selection to 1967 reviews.

To further dive in on papers investigating cancer therapies, we utilized many keywords derived from the National Cancer Institute’s “Types of Cancer Treatment” list. This approach was multi-faceted, with each set of keywords targeting a specific category of cancer therapy. For chemotherapy, we included terms like “chemotherapy”, “chemo”, and related variations. In the realm of hormone therapy, we searched for phrases such as “hormone therapy”, “hormonal therapy”, and similar terms. The keyword group for hyperthermia encompassed terms like “hyperthermia”, “microwave”, “radiofrequency”, and related technologies. For cancer vaccines, we included keywords such as “cancer vaccines”, “cancer vaccine”, and other related terms. The search for immune checkpoint inhibitors and immune system modulators was comprehensive, including terms like “immune checkpoint inhibitors”, “immunomodulators”, and various cytokines and growth factors. Lastly, our search for monoclonal antibodies and T-cell transfer therapy included relevant terms like “monoclonal antibodies”, “T-cell therapy”, “CAR-T”, and other related phrases. This keyword filtering leads to a pool of 352 reviews.

Then, we manually screened titles and abstracts, applying a rigorous classification and sorting methodology. The remaining papers were first categorized based on the type of cancer treatment they explored. We then organized these papers by their citation count to gauge their impact and relevance in the field. Our selection criteria aimed to enhance the quality and relevance of our final dataset. We prioritized papers that focused on the study of treatment effects, such as safety and efficacy, of various cancer interventions. We preferred studies that compared individual treatments against a control group, as opposed to those examining the effects of combined therapies (e.g., Therapy A + B vs. A only). To build a list of representative meta-analyses, we needed to ensure diversity in the target conditions under each treatment category.

Further, we favored studies that involved a larger number of individual studies, providing a broader base of evidence. However, we excluded network analysis studies and meta-analyses that focused solely on prognostic and predictive effects, as they did not align with our primary research focus. To maintain a balanced representation, we limited our selection to a maximum of three papers per treatment category. This process culminated in a final dataset comprising 100 systematic review papers.

### Prompt engineering

Prompting steers LLMs to conduct the target task without training the underlying LLMs. TrialMind proceeds clinical evidence synthesis in multiple steps associated with a series of prompting techniques.

The fundamental concept of in-context learning (ICL) is to enable LLMs to learn from examples and task instructions within a given context at inference time^8^. Formally, for a specific task, we define *T* as the task prompt, which includes the task definition, input format, and desired output format. During a single inference session with input *X*, the LLM is prompted with *P*(*T*, *X*), where *P*( ⋅ ) is a transformation function that restructures the task definition *T* and input *X* into the prompt format. The output $\hat{X}$ is then generated as $\hat{X}=\,{\text{LLM}}\,(P(T,X))$.

LLMs can produce hallucinations without high-quality evidence in their context. This issue can be mitigated through retrieval-augmented generation (RAG), which enhances LLMs by dynamically incorporating external knowledge into their prompts during generation^48^. We denote *R*_*K*_( ⋅ ) as the retriever that utilizes the input *X* to source relevant contextual information through semantic search. *R*_*K*_( ⋅ ) enables the dynamic infusion of tailored knowledge into LLMs at inference time.

Chain-of-though (CoT) guides LLMs in solving a target task in a step-by-step manner in one inference, hence handling complex or ambiguous tasks better and inducing more accurate outputs^49^. CoT employs the function *P*_CoT_( ⋅ ) to structure the task *T* into a series of chain-of-thought steps {*S*_1_, *S*_2_, …, *S*_*T*_}. As a result, we obtain $\{{\hat{X}}_{S}^{1},\ldots ,{\hat{X}}_{S}^{T}\}=\,\text{LLM}({P}_{{\rm{CoT}}}(T,X))$, all produced in a single inference session. This is rather critical when we aim to elicit the thinking process of LLM and urge it in self-reflection to improve its response. For instance, we may ask LLM to draft the initial response in the first step and refine it in the second.

Clinical evidence synthesis involves a multi-step workflow as outlined in the PRISMA statement^23^. It can be generally outlined as identifying and screening studies from databases, extracting characteristics and results from individual studies, and synthesizing the evidence. To enhance each step’s performance, task-specific prompts can be designed for an LLM to create an LLM-based module. This results in a chain of prompts that effectively addresses a complex problem, which we call LLM-driven workflow. Specifically, this approach breaks down the entire meta-analysis process into a sequence of *N* tasks, denoted as ${\mathcal{T}}=\{{T}_{1},\ldots ,{T}_{N}\}$. In the workflow, the output from one task, ${\hat{X}}_{n}$, serves as the input for the next, ${\hat{X}}_{n+1}=\,{\text{LLM}}\,(P({T}_{n},{\hat{X}}_{n}))$. This modular decomposition improves LLM performance by dividing the workflow into more manageable segments, increases transparency, and facilitates user interaction at various stages.

Incorporating these techniques, the formulation of TrialMind for any subtask can be represented as:

1 $${\hat{X}}_{n+1}=\,{\text{LLM}}\,(P({T}_{n},{X}_{n}),{R}_{K}({X}_{n})),\,\forall n=1,\ldots ,N,$$

where *R*_*K*_( ⋅ ) are optional.

### Implementation of TrialMind: study search

All experiments were run in Python v.3.9. Detailed software versions are: pandas v2.2.2; numpy v1.26.4; scipy v1.13.0; scikit-learn v1.4.1.post1; openai v1.23.6; langchain v0.1.16; boto3 v1.34.94; pypdf v4.2.0; lxml v5.2.1 and chromadb v0.5.0 with Python v.3.9.

We included GPT-4 and Sonnet in our experiments. GPT-4^50^ is regarded as a state-of-the-art LLM and has demonstrated strong performance in many natural language processing tasks (version: gpt-4-0125-preview). Sonnet^51^ is an LLM developed by Anthropic, representing a more lightweight but also very capable LLM (version: anthropic.claude-3-sonnet-20240229-v1:0 on AWS Bedrock). Both models support long context lengths (128K and 200K), enabling them to process the full content of a typical PubMed paper in a single inference session.

TrialMind processes research question inputs using the PICO (Population, Intervention, Comparison, Outcome) framework to define the study’s research question. In our experiments, the title of the target review paper served as the general description. Subsequently, we extracted the PICO elements from the paper’s abstract to detail the specific aspects of the research question.

TrialMind is tailored to adhere to the established guidelines^23^ in conducting literature searches and screening for clinical evidence synthesis. In the literature search stage, the key is formulating Boolean queries to retrieve a comprehensive set of candidate studies from databases. These queries, in general, are a combination of treatment and condition terms. However, direct prompting can yield low recall queries due to the narrow range of user inputs and the LLMs’ tendency to produce incorrect queries, such as generating erroneous MeSH (Medical Subject Headings) terms^9^. To address these limitations, TrialMind incorporates RAG to enrich the context with knowledge sourced from PubMed and employs CoT processing to facilitate a more exhaustive generation of relevant terms.

Specifically, the study search component has two main steps: initial query generation and then query refinement. In the first step, TrialMind prompts LLM to create the initial boolean queries derived from the input PICO to retrieve a group of studies (Prompt in Supplementary Fig. 6). The abstracts of these studies then enrich the context for refining the initial queries, working as RAG. In addition, we used CoT to enhance the refinement by urging LLMs to conduct multi-step reasoning for self-reflection enhancement (Prompt in Supplementary Fig. 7). This process can be described as

2 $$\{{\hat{X}}_{S}^{1},{\hat{X}}_{S}^{2},{\hat{X}}_{S}^{3}\}=\,{\text{LLM}}({P}_{{\rm{CoT}}}({T}_{{\rm{LS}}},X,{R}_{K}(X))),$$

where *X* denotes the input PICO; *R*_*K*_(*X*) is the set of abstracts of the found studies; *T*_LS_ is the definition of the query generation task for literature search. For the output, the first sub-step ${\hat{X}}_{S}^{1}$ indicates a complete set of terms identified in the found studies; the second ${\hat{X}}_{S}^{2}$ indicates the subset of ${\hat{X}}_{S}^{1}$ by filtering out the irrelevant; and the third ${\hat{X}}_{S}^{3}$ indicates the extension of ${\hat{X}}_{S}^{2}$ by self-reflection and adding more augmentations. In this process, LLM will produce the outputs for all three substeps in one pass, and TrialMind takes ${\hat{X}}_{S}^{3}$ as the final queries to fetch the candidate studies.

### Implementation of TrialMind: study screening

TrialMind follows PRISMA to take a transparent approach to study screening. It creates a set of eligibility criteria based on the input PICO as the basis for study selection (Prompt in Supplementary Fig. 8), produced by

3 $${\hat{X}}_{{\rm{EC}}}={\text{LLM}}(P({T}_{{\rm{EC}}},X)),$$

where ${\hat{X}}_{{\rm{EC}}}=\{{E}_{1},{E}_{2},\ldots ,{E}_{M}\}$ is the *M* generated eligibility criteria; *X* is the input PICO; and *T*_EC_ is the task definition of criteria generation. Users are given the opportunity to modify these generated criteria, further adjusting to their needs.

Based on ${\hat{X}}_{{\rm{EC}}}$, TrialMind embarks the parallel processing for the candidate studies. For *i*-th study *F*_*i*_, the eligibility prediction is made by LLM as (Prompt in Supplementary Fig. 9)

4 $$\{{I}_{i}^{1},\ldots ,{I}_{i}^{M}\}=\,{\text{LLM}}(P({F}_{i},X,{T}_{{\rm{SC}}},{\hat{X}}_{{\rm{EC}}})),$$

where *T*_SC_ is the task definition of study screening; *F*_*i*_ is the study *i*’s content; ${I}_{i}^{m}\in \{-1,0,1\},\forall m=1,\ldots ,M$ is the prediction of study *i*’s eligibility to the *m*-th criterion. Here, − 1 and 1 mean ineligible and eligible, 0 means uncertain, respectively. These predictions offer a convenient way for users to inspect the eligibility and select the target studies by altering the aggregation strategies. ${I}_{i}^{m}$ can be aggregated to offer an overall relevance of each study, such as ${\hat{I}}_{i}={\sum }_{m}{I}_{i}^{m}$. Users are also encouraged to extend the criteria set or block the predictions of some criteria to make customized rankings during the screening phase.

### Implementation of TrialMind: data extraction

Study data extraction is an open information extraction task that requires the model to extract specific information based on user inputs and handle long inputs, such as the full content of a paper. LLMs are particularly well-suited for this task because (1) they can be prompted for information extraction out of the box, and (2) they can take the whole publication content as input.

For the specified data fields to be extracted, TrialMind prompts LLMs to locate and extract the relevant information (Prompt in Supplementary Fig. 10). These data fields include (1) study characteristics such as study design, sample size, study type, and treatment arms; (2) population baselines; and (3) study findings. In general, the extraction process can be described as

5 $$\{{\hat{X}}_{\text{EX}}^{1},\ldots ,{\hat{X}}_{\text{EX}}^{K}\}=\,{\text{LLM}}(P(F,C,{T}_{{\rm{EX}}})),$$

where *F* represents the full content of a study; *T*_EX_ defines the task of data extraction; and *C* = {*C*_1_, *C*_2_, …, *C*_*K*_} comprises the series of data fields targeted for extraction. *C*_*k*_ is the user input natural language description of the target field, e.g., “the number of participants in the study". The input content *F* is segmented into distinct chunks, each marked by a unique identifier. The outputs, denoted as ${\hat{X}}_{\,\text{EX}\,}^{k}=\{{V}^{k},{B}^{k}\}$, include the extracted values *V* and the indices *B* that link back to their respective locations in the source content. Hence, it is convenient to check and correct mistakes made in the extraction by sourcing the origin. The extraction can also be easily scaled by making paralleled calls of LLMs.

### Implementation of TrialMind: result extraction

Our analysis indicates that data extraction generally performs well for study design and population-related fields; however, extracting study results presents challenges. Errors frequently arise due to the diverse presentation of results within studies and subtle discrepancies between the target population and outcomes versus those reported. For instance, the target outcome is the risk ratios (treatment versus control) regarding the incidence of adverse events (AEs), while the study reports AEs among many groups separately. Or, the target outcome is the incidence of severe AEs, which implicitly corresponds to those with grade III and more, while the study reports all grade AEs. To overcome these challenges, we have refined our data extraction process to create a specialized result extraction pipeline that improves clinical evidence synthesis. This enhanced pipeline consists of three crucial steps: (1) identifying the relevant content within the study (Prompt in Supplementary Fig. 11), (2) extracting and logically processing this content to obtain numerical values (Prompt in Supplementary Fig. 12), and (3) converting these values into a standardized tabular format (Prompt in Supplementary Fig. 13).

Steps (1) and (2) are conducted in one pass using CoT reasoning as

6 $$\{{\hat{X}}_{\,{\text{RE}}\,,S}^{1},{\hat{X}}_{\,\text{RE}\,,S}^{2}\}=\,{\text{LLM}}({P}_{{\rm{CoT}}}(X,O,F,{T}_{{\rm{RE}}})),$$

where *O* is the natural language description of the clinical endpoint of interest, and *T*_RE_ is the task definition of result extraction. In the outputs, ${\hat{X}}_{\,{\text{RE}}\,,S}^{1}$ represents the raw content captured from the input content *F* regarding the clinical outcomes; ${\hat{X}}_{\,{\text{RE}}\,,S}^{2}$ represents the elicited numerical values from the raw content, such as the number of patients in the group, the ratio of patients encountering overall response, etc. In step (3), TrialMind writes Python code to make the final calculation to convert ${\hat{X}}_{\,{\text{RE}}\,,S}^{2}$ to the standard tabular format.

7 $${\hat{X}}_{{\rm{RE}}}={\mathtt{exec}}(\,{\text{LLM}}(P(X,O,{T}_{{\rm{PY}}},{\hat{X}}_{\,{\text{RE}}\,,S}^{2})),{\hat{X}}_{\,{\text{RE}}\,,S}^{2}).$$

In this process, TrialMind adheres to the instructions in *T*_PY_ to generate code for data processing. This code is then executed, using ${\hat{X}}_{\,{\text{RE}}\,,S}^{2}$ as input, to produce the standardized result ${\hat{X}}_{{\rm{RE}}}$. An example code snippet made to do this transformation is shown in Supplementary Fig. 5. This approach facilitates verification of the extracted results by allowing for easy backtracking to ${\hat{X}}_{\,{\text{RE}}\,,S}^{1}$. Additionally, it ensures that the calculation process remains transparent, enhancing the reliability and reproducibility of the synthesized evidence.

### Experimental setup

In study search experiments, we assessed performance using the overall Recall, aiming to evaluate the effectiveness of different methods in identifying all relevant studies from the PubMed database using APIs^52^. For citation screening, we measured the performance using Recall@20 and Recall@50, which gauge how well the methods can prioritize target studies at the top of the list, thereby facilitating quicker decisions about which studies to include in evidence synthesis. We constructed the ranking candidate set for each review paper by initially retrieving studies through TrialMind, then refining this list by ranking the relevance of these studies to the target review’s PICO elements using OpenAI embeddings. The top 2000 relevant studies were kept. We then ensured all target papers were included in the candidate set to maintain the integrity of our ground truth data. The final candidate set was then deduplicated to be ranked by the selected methods.

In the criteria analysis experiment, we utilized Recall@200 to assess the impact of each criterion. This was done by first computing the relevance prediction using all eligibility predictions and then recalculating it without the eligibility prediction for the specific criterion in question. The difference in Recall@200 between these two relevance predictions, denoted as ΔRecall, indicates the criterion’s effect. A larger ΔRecall suggests that the criterion plays a more significant role in influencing the ranking results.

To evaluate the performance of data extraction, we measured the accuracy of the values extracted by TrialMind against the ground truth. We used the study characteristic tables from the review papers as our test set. Each table’s column names served as input field descriptions for TrialMind. We manually downloaded the full content for the studies listed in the characteristic table. To verify the accuracy of the extracted values, we enlisted three annotators who manually compared them against the data reported in the original tables.

We also measured the performance of result extraction using accuracy. The annotators were asked to carefully read the extracted results and compare them to the results reported in the original review paper. For the error analysis of TrialMind, the annotators were asked to check the sources to categorize the errors for one of the reasons: inaccurate, extraction failure, unavailable data, or hallucination. We designed a vanilla prompting strategy for GPT-4 and Sonnet models to set the baselines for the result extraction. Specifically, the prompt was kept minimal, as “Based on the {paper}, tell me the {outcome} from the input study for the population {cohort}", where {paper} is the placeholder for the paper’s content; {outcome} is the for the target endpoint; {cohort} is the for the target population’s descriptions, including conditions and characteristics. The responses from these prompts were typically in free text, from which annotators manually extracted result values to evaluate the baselines’ performance.

In evidence synthesis, we processed the input data using R and the meta package to make the forest plots and the pooled results based on the standardized result values. This is for both TrialMind and the baselines. Nonetheless, for the baseline, the annotators also need to manually extract the result values and standardize the values to make them ready for meta-analysis, which forms the GPT-4+Human baseline in the experiments.

We engaged two groups of annotators for our evaluation: (1) three computer scientists with expertise in AI applications for medicine, and (2) five medical doctors to assess the generated forest plots. Each annotator was asked to evaluate five review studies. For each review, we randomly presented forest plots generated by both the baseline and TrialMind. The annotators were required to determine how closely each generated plot aligned with a reference forest plot taken from the target review paper. Additionally, they were asked to judge which method, the baseline or TrialMind, produced better results in a win/lose assessment. Supplementary Fig. 3 demonstrates the user interface for this study, which was created with Google Forms.

## Supplementary information

Supplementary Information

## Data Availability

The TrialReviewBench dataset for study search, citation screening, and data extraction tasks can be accessed at https://huggingface.co/datasets/zifeng-ai/TrialReviewBench.

## Code availability

TrialMind can be accessed at https://github.com/RyanWangZf/TrialMind-SLR, including the source code for implementing TrialMind’s study search, study screening, data extraction, and result extraction components. The interactive web-based demo of TrialMind is hosted at https://www.trialmindapis.com/api/systematic-literature-review. Access is available upon request by contacting the corresponding author.
