# Supplementary material for: Accelerating clinical evidence synthesis with large language models
Source: NPJ Digit Med. 2025 Aug 8;8:509. doi: 10.1038/s41746-025-01840-7 (PMC12331930; doi:10.1038/s41746-025-01840-7)
Supplement: Supplementary file 1 — Supplementary Information [file 41746_2025_1840_MOESM1_ESM.pdf]

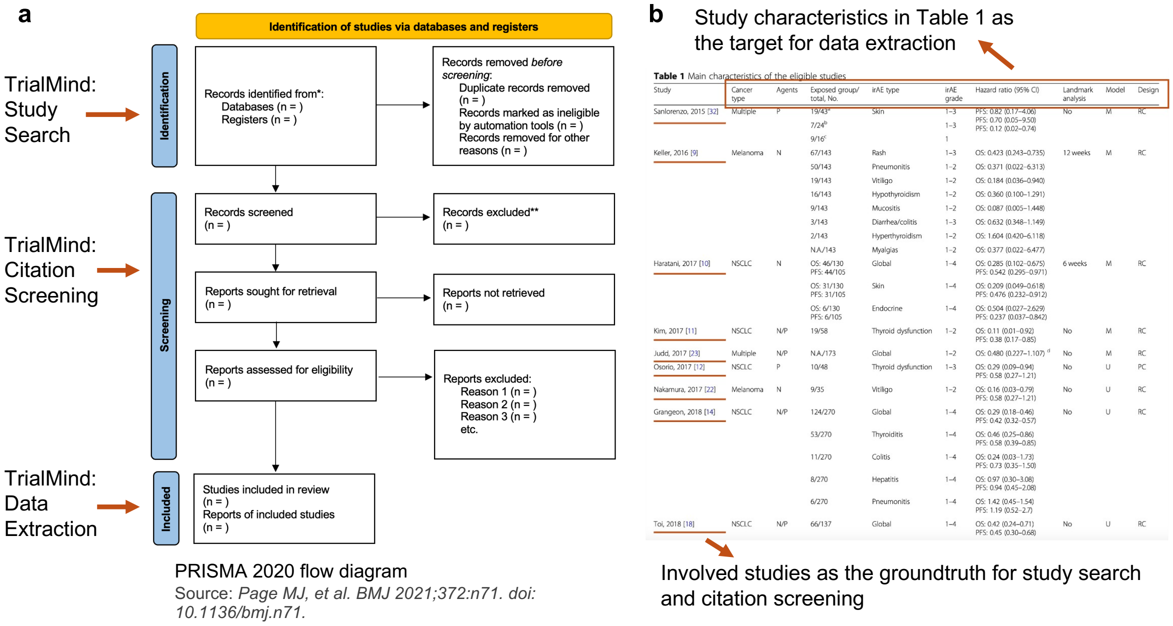

Supplementary Figure 1: **TrialMind streamlines systematic reviews and aligns with PRISMA for evaluation.** **a**, TrialMind aligns with PRISMA's systematic review process by streamlining key steps: (1) Identification, generating search terms from PICO elements for comprehensive study retrieval; (2) Screening, creating and applying inclusion/exclusion criteria to assess study eligibility, and (3) Inclusion, extracting target data fields, providing sources, and aggregating study outcomes via meta-analysis. This design enables seamless integration into the systematic literature review workflow while supporting human-AI collaboration. **b**, TrialMind 's performance is evaluated using published systematic reviews as test sets. Given a review paper, included studies are extracted as target studies for identification and screening, while target data from its "Table 1" serve as ground truth for data extraction. This setup ensures evaluation accuracy and alignment with PRISMA practices.

a

Topic	PMID	GPT-4	TrialMind	$\Delta Recall^*$
Blood	31710397	1.00	1.00	0.00
Circulatory	17253464	0.00	1.00	1.00
Digestive	23450555	0.00	1.00	1.00
Eye	15674896	0.00	1.00	1.00
Genitourinary	10796413	0.33	0.56	0.23
Health	18843733	0.00	0.80	0.80
Infectious	12535504	0.33	1.00	0.67
Injury	16625622	0.00	1.00	1.00
Metabolic	21735425	0.86	0.86	0.00
Musculoskeletal	19160181	0.00	0.60	0.60
Neoplasms	23235672	0.00	0.57	0.57
Nervous	25756660	0.00	0.95	0.95
Perinatal	27281496	0.00	1.00	1.00
Pregnancy	23450559	0.07	0.93	0.86
Respiratory	20464732	0.00	1.00	1.00
Skin	16625648	0.00	0.57	0.57

*The difference of TrialMind's Recall and GPT-4's Recall in study search tasks; Green: TrialMind is better; Red: GPT-4 is better.

b

Topic	PMID	Dense	TrialMind	$\Delta Recall^*$
Blood	31710397	1.00	1.00	0.00
Circulatory	17253464	1.00	1.00	0.00
Digestive	23450555	1.00	1.00	0.00
Eye	15674896	0.82	0.84	0.02
Genitourinary	10796413	1.00	1.00	0.00
Health	18843733	0.92	1.00	0.08
Infectious	12535504	1.00	1.00	0.00
Injury	16625622	1.00	1.00	0.00
Metabolic	21735425	1.00	1.00	0.00
Musculoskeletal	19160181	0.60	0.72	0.12
Neoplasms	23235672	0.43	0.57	0.14
Nervous	25756660	1.00	1.00	0.00
Perinatal	27281496	1.00	1.00	0.00
Pregnancy	23450559	0.14	0.36	0.21
Respiratory	20464732	0.43	0.63	0.20
Skin	16625648	0.86	0.71	-0.14

*The difference of TrialMind's Recall and Dense method's Recall in citation screening tasks; Green: TrialMind is better; Red: Dense method is better.

Supplementary Figure 2: **Pilot study of `TrialMind` across broad therapeutic areas.** **a**, We evaluate the Recall of GPT-4-generated search queries and `TrialMind`-generated search queries across systematic reviews in different therapeutic areas categorized by ICD-10-CM codes. Rows where `TrialMind` performs at least as well as GPT-4 are highlighted in green. **b**, We compare `TrialMind` with the dense method for their study eligibility ranking performance by Recall@50. Rows, where `TrialMind` outperforms the dense method, are highlighted in green, while those where it underperforms are highlighted in red.

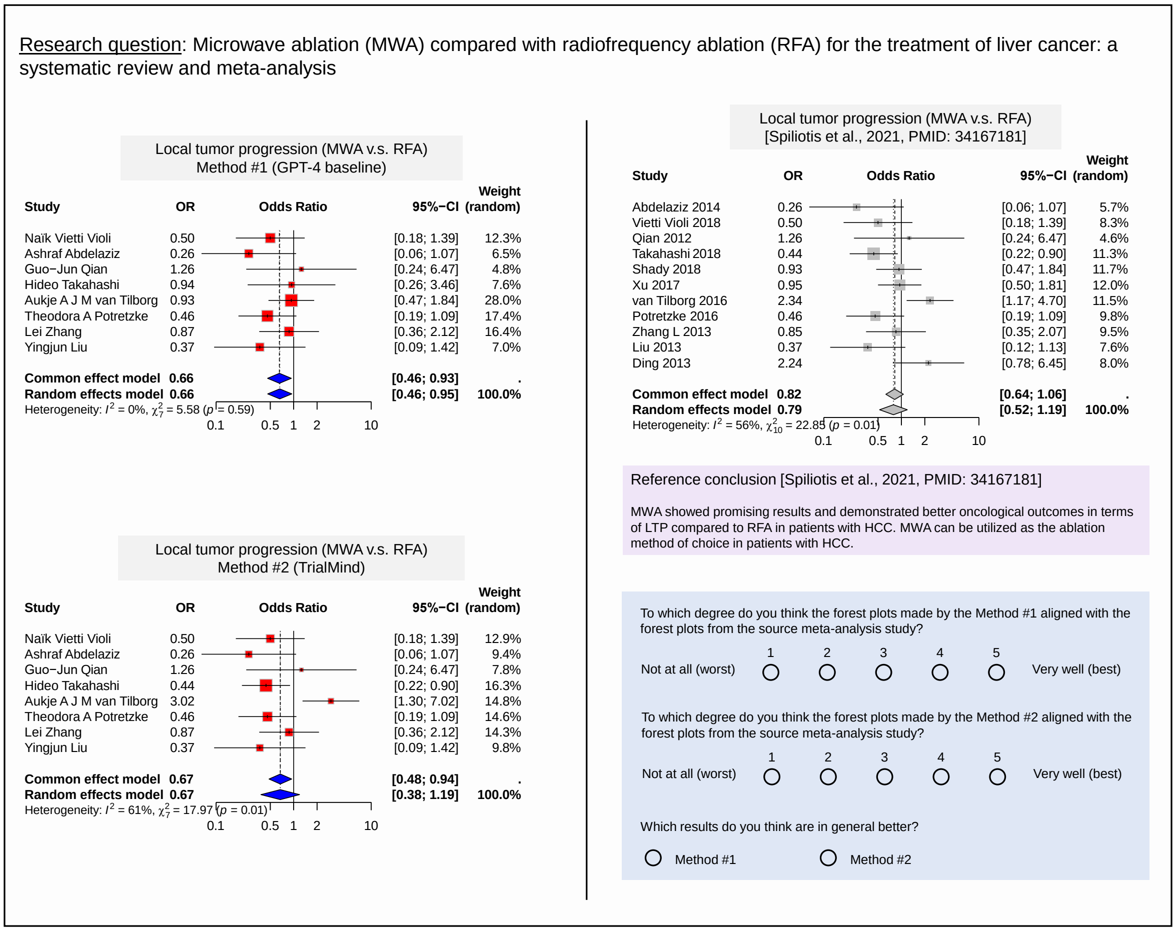

Supplementary Figure 3: **Comparative evaluation of clinical evidence synthesis using forest plots**. Study design for comparative evaluation of clinical evidence synthesis between baseline and `TrialMind` approaches. Annotators evaluated forest plots generated by both methods on a 5-point scale (1-5) and provided a binary preference judgment to determine superior performance. Method comparison was conducted through a systematic assessment of forest plot quality and comparative effectiveness analysis.

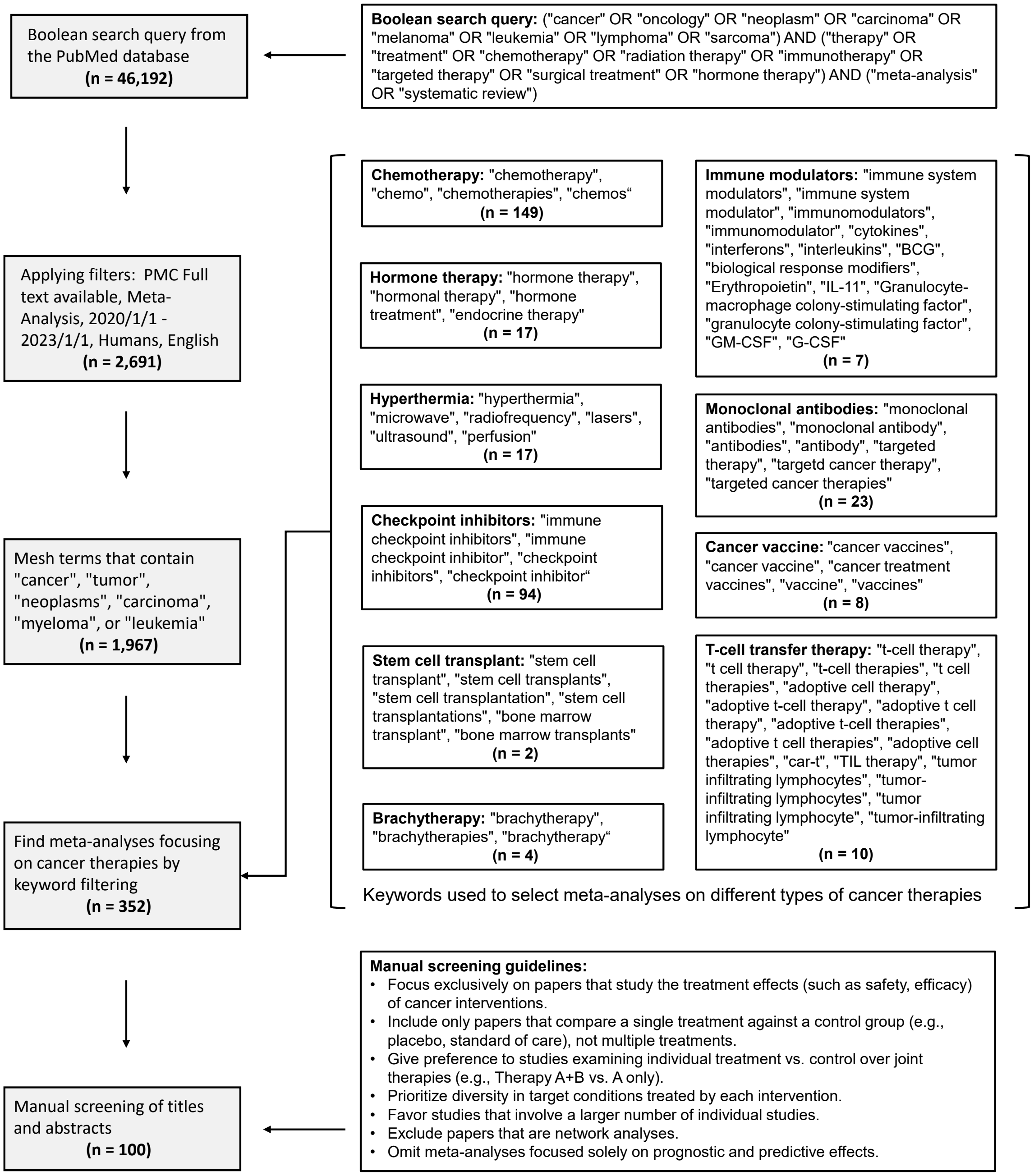

Supplementary Figure 4: **Building `TrialReviewBench` benchmark from the literature**. The workflow for selecting meta-analyses on cancer therapies from PubMed, including Boolean search queries, keyword filtering, and manual screening based on predefined criteria.

Generated Result Extraction Code

```python
import pandas as pd
import numpy as np

def classify_arms(df: pd.DataFrame) -> pd.DataFrame:
    # Assuming the 'Group' column already exists and classifies arms as 'Experimental' or 'Control'
    # If not, code to classify arms based on the research question would be added here
    return df

def consolidate_data(df: pd.DataFrame) -> pd.DataFrame:
    # Consolidate the data for each group
    # Calculate the number of participants who do not experience the event
    df['No event'] = df['N'] - df['Complete Response']

    # Group by 'Group' and sum up the 'Complete Response', 'No event', and 'N'
    consolidated = df.groupby('Group').agg({
        'Complete Response': 'sum',
        'No event': 'sum',
        'N': 'sum'
    }).reset_index()

    # Rename columns to match the target output
    consolidated.rename(columns={
        'Group': 'Study',
        'Complete Response': 'Event',
        'N': 'Total'
    }, inplace=True)

    # If no control group is present, add a row for control with zeros
    if 'Control' not in consolidated['Study'].values:
        consolidated = pd.concat([consolidated, pd.DataFrame({
            'Study': ['Control'],
            'Event': [0],
            'No event': [0],
            'Total': [0]
        })], ignore_index=True)

    return consolidated

def calculate_statistics(df: pd.DataFrame) -> pd.DataFrame:
    # This function is a placeholder for any statistical calculations that might be needed
    # For this example, no additional statistics are calculated
    return df

# Assuming 'df' is the dataframe containing the raw data
# df = ...

# Run these functions in sequence to get the final dataframe
df = classify_arms(df)
df = consolidate_data(df)
df = calculate_statistics(df)
```

Make a classification of the input data

Consolidate the raw data to the target outcome

Make the final calculation to get the standardized outcomes

Supplementary Figure 5: **`TrialMind` synthesizes code to compute trial results**. Here is an example Python code made by `TrialMind` when converting the extracted result values to standardized tabular form.

Prompt for initial query generation

You are a clinical specialist. You are conducting a clinical study meta-analysis.
The research is defined by the following PICO elements:
P (Patient, Problem or Population): {P}
I (Intervention): {I}
C (Comparison): {C}
O (Outcome): {O}

Task
Your task is to identify the primary clinical term(s) in this research.
The clinical terms should be specific medical conditions, treatments, or procedures.
General terms such as 'patients', or 'therapy' should not be included.

Reply Format
You should only reply with 1~3 primary term. Your output should be in JSON format, like this:

```
{{
    "terms": ["term1", "term2", "term3"]
}}
```

Supplementary Figure 6: **Prompt for study search**. Prompt for generating initial search queries in the literature search.

Prompt for query expansion and refinement

```
## Background

You are a clinical specialist. You are conducting a clinical meta-analysis.
The research is defined by the following PICO elements:
P (Patient, Problem or Population): {P}
I (Intervention): {I}
C (Comparison): {C}
O (Outcome): {O}

## Reference

You've already gathered these related papers:
{pubmed_reference_text}

## Task

Your task is to further your literature search by these 3 steps:

### Step 1
Extract related term in the reference papers.
Provide three lists of query terms: TREATMENTS, CONDITIONS, and OUTCOMES.

CONDITIONS: words about any conditions or disease that is related to this meta-analysis (referring to Problem section)
TREATMENTS: primary related clinical terms/keywords showed in these reference papers (referring to Intervention section)
OUTCOMES: clinical endpoints or outcome measurements that are related to this meta-analysis (referring to Outcome section)

### Step 2
Double-check these query terms, remove the terms that is not directly related to the PICO elements of this research.
Provide three lists of refined core terms: CORE_CONDITIONS, CORE_TREATMENTS, and CORE_OUTCOMES.

CORE_CONDITIONS: refined terms of conditions or disease
CORE_TREATMENTS: refined terms of primary related clinical terms/keywords
CORE_OUTCOMES: refined terms of clinical endpoints or outcome measurements

### Step 3
To expand the scope of query term searches, please extend each query term by:
1. Synonyms and other names/forms;
2. Possible abbreviations or full forms;
3. Split into elements for compound phrases.
Provide three lists of expanded query terms: EXPAND_CONDITIONS, EXPAND_TREATMENTS, EXPAND_OUTCOMES.

EXPAND_CONDITIONS: expanded terms of conditions or disease
EXPAND_TREATMENTS: expanded terms of primary related clinical terms/keywords
EXPAND_OUTCOMES: expanded terms of clinical endpoints or outcome measurements


## Reply format
There should be no overlap between each pair of lists

Your reply should be in a format like:

{{

"step 1": {{
   "CONDITIONS": [condition1, condition2, ..] \\ (~10 items)
   "TREATMENTS": [term1, term2 .. ] \\ (~10 items)
   "OUTCOMES": [outcome1, outcome2, ..] \\ (~10 items)
}},

\\ Refine according to P (Patient, Problem or Population): {P} and I (Intervention): {I} and O (Outcome): {O}
"step 2": {{
   "CORE_CONDITIONS": [condition1, condition2, ..] \\ (~5 items)
   "CORE_TREATMENTS": [term1, term2, .. ] \\ (~5 items)
   "CORE_OUTCOMES" : [outcome1, outcome2 ..] \\ (~5 items)
}},

\\ Augmentation
"step 3": {{
   "EXPAND_CONDITIONS": [condition1, condition2, ..]  \\ (~10 items)
   "EXPAND_TREATMENTS": [term1, term2 ..] \\ (~10 items)
   "EXPAND_OUTCOMES": [outcome1, outcome2 ..] \\ (~10 items)
   }}
}}
```

Supplementary Figure 7: **Prompt for study search**. Prompt for expanding and refining the initial search queries in the literature search.

Prompt for study eligibility criteria generation

You are a clinical specialist. You are conducting a clinical meta-analysis.
The research is defined by the following PICO elements:
P (Patient, Problem or Population): {P}
I (Intervention): {I}
C (Comparison): {C}
O (Outcome): {O}

Task
Your task is to design the eligibility criteria for selecting studies for this meta-analysis study following these 3 steps:

Step 1
Based on the PRISMA guidelines and the PICO elements of this research, please identify five eligibility criteria for the studies to be included in the meta-analysis. Provide a rationale for each criterion.

ELIGIBILITY_ANALYSIS: your items and reasons here...

Step 2
Next, create {num_title_criteria} binary questions that will help you select studies based on their titles.
These questions should be designed so that a "YES" answer indicates the study meets the criteria, while a "NO" answer means it doesn't.
The information required to answer these questions should be general and easily found in the study title.

TITLE_CRITERIA n: ...

Step 3
Finally, develop {num_abstract_criteria} more binary questions to further filter the studies based on their content.
These questions should also be designed for a "YES" or "NO" answer,
but the information required to answer them will be more detailed and is expected to be found within the main content of the study.

CONTENT_CRITERIA n: ...

Reply Format
You should reply in a format like:

```
{{
   "ELIGIBILITY_ANALYSIS": ["rationale1", "rationale12", ...] \\ the bullet points of your analysis
   "TITLE_CRITERIA": ["criterion1", "criterion2", "..."] \\ the {num_title_criteria} binary title-based criteria
   "CONTENT_CRITERIA": ["criterion1", "criterion2", "..."] \\ the {num_abstract_criteria} binary content-based criteria
}}
```

Supplementary Figure 8: **Prompt for study screening**. Prompt for study eligibility criteria generation in the literature screen.

Prompt for study eligibility assessment

```
# CONTEXT #
You are a clinical specialist tasked with assessing research papers for inclusion in a
meta-analysis based on specific eligibility criteria.

# OBJECTIVE #
Evaluate each criterion of a given paper to determine its eligibility for inclusion in the
meta-analysis. Provide a list of decisions ("YES", "NO", or "UNCERTAIN") for each
eligibility criterion. You must deliver exactly {num_criteria} responses.

# IMPORTANT NOTE #
If the information within the provided paper content is insufficient to conclusively
evaluate a criterion, you must opt for "UNCERTAIN" as your response. Avoid making
assumptions or extrapolating beyond the provided data, as accurate and reliable
responses are crucial, and fabricating information (hallucinations) could lead to
serious errors in the meta-analysis.

# PICO FRAMEWORK #
- P (Patient, Problem or Population): {P}
- I (Intervention): {I}
- C (Comparison): {C}
- O (Outcome): {O}

# PAPER DETAILS #
- Provided Paper: {paper_content}

# EVALUATION CRITERIA #
- Number of Criteria: {num_criteria}
- Criteria for Inclusion: {criteria_text}

# RESPONSE FORMAT #
You are required to output a JSON object containing a list of decisions for each of
the {num_criteria} eligibility criteria. Each decision should directly correspond to one
of the criteria and be listed in the order they are presented. Ensure to use
"UNCERTAIN" wherever the paper does not explicitly support a "YES" or "NO"
decision.
For example:
'''json
{{
   "evaluations": ["YES", "NO", "UNCERTAIN", "YES", "YES", ...] \\ List of
{num_criteria} decisions
}}
'''
```

Supplementary Figure 9: **Prompt for study screening**. Prompt for study eligibility assessment in the literature screen.

Prompt for study characteristics extraction

```
You are now the following python function: ```
def extract_fields_from_input_study(inputs: Dict[str, Any]) -> str:
   \"\"\"
   This function is tasked with analyzing clinical trial study reports or papers to extract specific information as
structured data
   and provide citations for the extracted information.
   The user will provide a list of fields they are interested in, along with a natural language description for each
field to guide you on what content to look for and from which parts of the report to extract it.

   IMPORTANT:
   For each field described by the user, you need to:
   1. Identify and extract the relevant information from the report based on the provided description.
   2. Generate a field name that accurately represents the content of the field based on its description.
   3. Structure the extracted information into a standard format whenever possible (e.g., integer, numerical values,
dates, keywords, list of terms).
      If standardization is not possible, the information should be presented in text format.
      If the field is not found in the report, the extracted value should be "NP".
   4. Provide a reference to the document ID from which this information was extracted.
      This citation id should be restricted to be integers only.
      You should NOT cite more than three sources for a single field.
      You should try your best to provide the most relevant and specific citation for each field.
      If two or more sources are equally relevant, you can just cite one of them.

   The function returns a string representing a dictionary with each key representing a field and its extracted
value. The format should be as follows:

   Returns: A syntactically correct JSON string representing a list of dictionary with three keys: name, value, and
source_id.
      Format:
      ```json
      [
         {{
            "name":  \\ str, length <= 25 tokens
            "value":  \\ str, length <= 25 tokens
            "source_id":  \\ list[int], length <= 3 ids
         }},
         {{
            "name":, \\ str, length <= 25 tokens
            "value":  \\ str, length <= 25 tokens
            "source_id":  \\ list[int], length <= 3 ids
         }},
         ...
      ]
      ```
   \"\"\"
```
Respond exclusively with the generated JSON string wrapped ```json and ```.

# User provided inputs
paper_content = \"\"\"{paper_content}\"\"\"
fields = \"\"\"{fields}\"\"\"

inputs = {{
   "paper_content": paper_content,
   "fields": fields
}}
```

Supplementary Figure 10: **Prompt for data extraction**. Prompt for study characteristics extraction in the data extraction.
```

Prompt for initial result extraction and localization

```
You are now the following python function: ```
def locate_evidence_in_study_about_the_request_for_results(inputs: Dict[str, Any]) -> str:
    \"\"\"
    This function is tasked with analyzing clinical trial study reports or papers to extract specific information as structured data.

    Task Instructions:
    1. Review the clinical trial paper, paying close attention to the sections discussing results related to the "{target_outcome}" for the
defined cohort "{cohort}".
    2. Summarize the findings for each cohort, emphasizing the collective data and general trends observed. Individual patient data
should only be mentioned if highlighted as a significant exception or case study in the paper.
    3. Present your summary in a table format with the following columns:
    - 'Group Name': Name of the cohort.
    - 'Number of Patients': Total participants in the cohort.
    - 'Specified Outcome Measure': Key findings and metrics related to the "{target_outcome}".
    Include aggregate values such as percentages, mean values, and other statistical summaries that reflect the overall results for the
group.
    Must contain quantitative data, such as hazard ratios, odds ratios, mean differences, count of events, etc.
    Do not include qualitative or descriptive data that cannot be quantified, such as "statistically significant improvement" without
specific values.

    Here are the definitions of some common outcome measurements:
    - overall survival/progression-free survival/etc.: usually defined by the hazard ratio or odds ratio, which is the ratio of hazard rate or
odds of an event occurring in the treatment group to that in the control group.
        it can also be expressed as the number of events in the target group of patients.
    - toxicity/adverse events/etc.: defined by the rate of occurrence of adverse events in the target groups of patients.
    - objective response rate/overall response/etc.: defined by the proportion of patients who respond to the treatment in the target
groups, or number of patients with complete or partial response.
    - disease control rate/relapse rate/etc.: defined by the proportion of patients who have stable disease or better in the target groups,
or number of patients with disease control.
        it can also be expressed by the number of patients who have disease progression, so the disease control rate is 1 minus the
progression rate.

    Returns:
        A str representing a list of dictionary with three keys: Group Name, N, and Results.
        Group Name: str - name of the cohort
        N: int - number of participants in the cohort
        Results: str - key findings and metrics related to the outcome measure, must be quantitative and concise
            adhere to the input paper content.
        Example format:
        ```json
        [
            {{
                "Group Name": str, \\ the name of the cohort
                "N": int, \\ the number of participants in the cohort
                "Results": str \\ key findings and metrics related to the outcome measure, must be quantitative and concise (<= 50 tokens)
            }},
            {{
                ...
            }},
            ...
        ]
        ```
    \"\"\"
```
Respond exclusively with the generated JSON string wrapped ```json and ```.

# User provided inputs
paper_content = \"\"\"{paper_content}\"\"\"
cohort = \"\"\"{cohort}\"\"\"
target_outcome = \"\"\"{target_outcome}\"\"\"

inputs = {{
    "paper_content": paper_content,
    "cohort": cohort,
    "target_outcome": target_outcome
}}
```

Supplementary Figure 11: **Prompt for trial result extraction**. Prompt for the initial result extraction in the evidence synthesis.
```

Prompt for the study result table formatting

```
You are now the following python function: ```
def format_study_result_table(inputs: Dict[str, Any]) -> str:
    \"\"\"
    This function is used to transform raw data (with the content described in texts) from a clinical study paper into a structured table format.

    IMPORTANT: Organize the extracted data into a structured table format. Ensure that each column represents a crucial numerical data
point necessary for meta-analysis. This may include participant numbers (N), measurable outcomes,
        and other quantifiable metrics related to the intervention or comparator.

    IMPORTANT: The input groups are not mutually exclusive, you need to decide whether to combine them or
        select the eligible groups based on the research question.

    IMPORTANT: Drop the group if no outcome value is provided.

    The function returns a string representing a list of dictionary, each dictionary has three keys:
    ```json
    [
        {{
            Group: str \\ the name of the group
            N: int \\ the number of participants in the group
            Outcome Value: float or int \\ the outcome value for the group, must be float or int values
        }},
        {{
            ...
        }},
        ...
    ]
    ```

    Example output 1:
    ```json
    [
        {{
            "Group": "Patient with irAEs",
            "N": 100,
            "Hazard Ratio": 0.5,
        }},
        {{
            "Group": "Patient without irAEs",
            "N": 150,
            "Hazard Ratio": 0.3,
        }},
    ]
    ```
    [… some more examples]

    Returns:
        A str representing a list of dictionary with three keys: Groups, N, and Outcome.
        Groups: str - the name of the group
        N: int - the number of participants in the group
        {outcome}: float or int - the outcome value for the group, must be float or int
    \"\"\"
```
Respond exclusively with the generated JSON string wrapped ```json and ```.

# User provided inputs
results = \"\"\"{results}\"\"\"
outcome = \"\"\"{outcome}\"\"\"
intervention = \"\"\"{intervention}\"\"\"
comparator = \"\"\"{comparator}\"\"\"
population = \"\"\"{population}\"\"\"

inputs = {{
    "results": results,
    "outcome": outcome,
    "intervention": intervention,
    "comparator": comparator,
    "population": population
}}
```

Supplementary Figure 12: **Prompt for trial result extraction**. Prompt for the result formatting in the evidence synthesis.
```

Prompt for the study result standardization

```
You are now the following python function: ```
def generate_continuous_elegant_python_code(inputs: Dict[str, Any]) -> str:
    \"\"\"
    This function is used to generate python code to transform raw data into a structured format according to a specified schema.
    The raw data is collected from a clinical study paper and needs to be organized to facilitate a meta-analysis study.
    The target meta-analysis is driven by a specific research question, defined by the Population, Intervention, Comparator, and Outcome
(PICO) elements.

    The function takes a dictionary of `inputs` as an argument, which contains the following keys:
    - 'research_question': contains the population, intervention, comparator, and outcome (PICO) elements of the targeted meta-analysis.
        Based on the research question's intervention and comparator, you need to classify which arms in the raw data belong to the targeted
        experimental and control groups.
        Based on the research question's population, you need to consider which parts of the observations in the raw data are relevant to the
targeted meta-analysis.
    - 'raw_data': this is the markdown formatted input dataframe that contains the raw data to be transformed.
    - 'desc': the description of the target dataframe the generated code needs to produce.
    - 'target_output': the generated code needs produce a dataframe follows the structure of this target_output.

    IMPORTANT: 'raw_data' does **NOT** need to be parsed or loaded into a dataframe. In your generated code,
        just use `df` to represent the dataframe that contains the raw data.

    IMPORTANT: NaN values should be treated with caution. If the raw data contains NaN values, ensure that your code handles them
appropriately,
        either by removing the row with any NaN values or by filling all NaN with zeros.

    IMPORTANT: Never use `pd.DataFrame.append` to add data to pd.DataFrame. This method has been deprecated, and it is
recommended to use `pd.concat` instead.
        For example, to add a new row to a dataframe, you can use `df = pd.concat([df, new_row], ignore_index=True)`
        Never use `df.append(new_row, ignore_index=True)`

    The function returns a string of raw Python code, wrapped within <code> and </code> tags. For example:
    <code>
    # df
    # Your generated python code here...
    </code>

    You should implemented three functions in your code, each of which takes a dataframe as input and returns a dataframe as output:
    <code>
    # Dataframe definition should be ignored and not show again
    # df = ...

    def classify_arms(df: pd.DataFrame):
        # you need to define a new column in the dataframe to indicate which group each arm belongs to
        # each arm should be classified as either experimental or control
        # e.g., `df['Group'] = ['Experimental', 'Control', 'Experimental', ...]`
        return df

    def consolidate_data(df: pd.DataFrame):
        # consolidate the data for each group
        return df

    def calculate_statistics(df: pd.DataFrame):
        # calculate the statistics for each group
        return df

    # run these functions in sequence to get the final dataframe
    df = classify_arms(df)
    df = consolidate_data(df)
    df = calculate_statistics(df)
    </code>

    Returns:
        Executable Python code that will be used to transform the raw data into the structured format.
    \"\"\"
```
Respond exclusively with the generated code wrapped <code></code>. Ensure that the code you generate is executable Python code that
can be run directly in a Python environment, requiring no additional string encapsulation.

# User provided inputs
research_question = \"\"\"Population: {population}, Intervention: {intervention}, Comparator: {comparator}, Outcome: {outcome}\"\"\"
raw_data = \"\"\"{raw_data}\"\"\"
desc = \"\"\"{desc}\"\"\"
target_output = \"\"\"{target_output}\"\"\"

inputs = {{
    "research_question": research_question,
    "raw_data": raw_data,
    "desc": desc
    "target_output": target_output
}}
```

Supplementary Figure 13: **Prompt for trial result extraction**. Prompt for the result standardization in the evidence synthesis.
```

Supplementary Movie 1: Automated systematic reviews with `TrialMind`. We have attached a demonstration video showing how `TrialMind` can be interacted via a web-based platform for medical users. It allows users to sift through the process of study search, screening, and data extraction tasks to conduct a systematic literature review following the PRISMA statement. The video is available at https://www.youtube.com/watch?v=5VKt_s4X5MO&ab_channel=AIforhealth.
